# Management of neutropenic patients in the intensive care unit (NEWBORNS EXCLUDED) recommendations from an expert panel from the French Intensive Care Society (SRLF) with the French Group for Pediatric Intensive Care Emergencies (GFRUP), the French Society of Anesthesia and Intensive Care (SFAR), the French Society of Hematology (SFH), the French Society for Hospital Hygiene (SF2H), and the French Infectious Diseases Society (SPILF)

**DOI:** 10.1186/s13613-016-0189-6

**Published:** 2016-09-15

**Authors:** David Schnell, Elie Azoulay, Dominique Benoit, Benjamin Clouzeau, Pierre Demaret, Stéphane Ducassou, Pierre Frange, Matthieu Lafaurie, Matthieu Legrand, Anne-Pascale Meert, Djamel Mokart, Jérôme Naudin, Frédéric Pene, Antoine Rabbat, Emmanuel Raffoux, Patricia Ribaud, Jean-Christophe Richard, François Vincent, Jean-Ralph Zahar, Michael Darmon

**Affiliations:** 1 Angouleme Hospital, Angoulême, France; 2 Saint-Louis University Hospital, Paris, France; 3 Intensive Care Unit, Ghent University Hospital, Ghent, Belgium; 4 Medical Intensive Care Unit, Pellegrin University Hospital, Bordeaux, France; 5 Paediatric Intensive Care Unit, Centre Hospitalier Chrétien, Liège, Belgium; 6 Pediatric Hematological Unit, Bordeaux University Hospital, Bordeaux, France; 7 Microbiology Laboratory & Pediatric Immunology - Hematology Unit, Necker University Hospital, Paris, France; 8 Department of Infectious Diseases, Saint-Louis University Hospital, Paris, France; 9 Surgical ICU and Burn Unit, Saint-Louis University Hospital, Paris, France; 10 Thoracic Oncology Department and Oncologic Intensive Care Unit, Institut Jules Bordet, Brussels, Belgium; 11 Polyvalent Intensive Care Unit, Department of Anesthesiology and Critical Care, Institut Paoli Calmette, Marseille, France; 12 Pediatric ICU, Robert Debré University Hospital, Paris, France; 13 Cochin University Hospital Hospital, Paris, France; 14 Respiratory Intensive Care Unit, Cochin University Hospital Hospital, Paris, France; 15 Department of Hematology, Saint-Louis University Hospital, Paris, France; 16 Department of Stem Cell Transplantation, Saint-Louis University Hospital, Paris, France; 17 Croix Rousse University Hospital, Lyon, France; 18 Montfermeil Hospital, Montfermeil, France; 19 Infection Control Unit, Angers University Hospital, Angers, France; 20 University Hospital, Saint-Etienne, France; 21 Medical-Surgical Intensive Care Unit, Saint-Etienne University Hospital, Avenue Albert Raymond, 42270 Saint-Etienne, Saint-Priest-En-Jarez France

**Keywords:** Neutropenia, Intensive care units, Guidelines, Prognosis, Antibiotics, Isolation

## Abstract

Neutropenia is defined by either an absolute or functional defect (acute myeloid leukemia or myelodysplastic syndrome) of polymorphonuclear neutrophils and is associated with high risk of specific complications that may require intensive care unit (ICU) admission. Specificities in the management of critically ill neutropenic patients prompted the establishment of guidelines dedicated to intensivists. These recommendations were drawn up by a panel of experts brought together by the French Intensive Care Society in collaboration with the French Group for Pediatric Intensive Care Emergencies, the French Society of Anesthesia and Intensive Care, the French Society of Hematology, the French Society for Hospital Hygiene, and the French Infectious Diseases Society. Literature review and formulation of recommendations were performed using the Grading of Recommendations Assessment, Development and Evaluation system. Each recommendation was then evaluated and rated by each expert using a methodology derived from the RAND/UCLA Appropriateness Method. Six fields are covered by the provided recommendations: (1) ICU admission and prognosis, (2) protective isolation and prophylaxis, (3) management of acute respiratory failure, (4) organ failure and organ support, (5) antibiotic management and source control, and (6) hematological management. Most of the provided recommendations are obtained from low levels of evidence, however, suggesting a need for additional studies. Seven recommendations were, however, associated with high level of evidences and are related to protective isolation, diagnostic workup of acute respiratory failure, medical management, and timing surgery in patients with typhlitis.

**Electronic supplementary material:**

The online version of this article (doi:10.1186/s13613-016-0189-6) contains supplementary material, which is available to authorized users.

## Background

Neutropenia is defined by an absolute count of polymorphonuclear neutrophils (PMNs) less than 1500/mm^3^ [1]. Infections are the main complication of this condition, and the risk is higher when neutropenia is profound (PMN < 500/mm^3^) and/or prolonged (more than 7 days) [2, 3]. The diagnosis of these infections is hampered by the relative paucity of symptoms, leading to severe status in cases of delayed diagnosis [2, 4]. In intensive care unit (ICU), up to 2.4 % of the overall population [5], 8.6 % of patients with sepsis or septic shock [6], and 28.6 % of hematological patients [7] will experience neutropenia or leucopenia during their ICU stay.

Management strategies for neutropenic patients in the ICU often rely on low-grade evidence derived from an extensive but sometimes contradictory medical literature partly ascribable to small single-center observational cohort studies, variability regarding centers experience and volume of critically ill cancer patients, and relatively old studies. The management specificities of these patients in the ICU prompted the establishment of guidelines dedicated to intensivists.

## Methods

These recommendations were drawn up by a panel of experts brought together by the French Intensive Care Society (SRLF) in collaboration with scientific societies in disciplines that contribute to the management of neutropenic patients: the French Group for Pediatric Intensive Care Emergencies (GFRUP), the French Society of Anesthesia and Intensive Care (SFAR), the French Society of Hematology (SFH), the French Society for Hospital Hygiene (SF2H), and the French Infectious Diseases Society (SPILF). The organizing committee appointed a coordinator, who selected the SRLF experts. Each associated scientific society selected its experts. The coordinator first defined the field to be covered and proposed experts to be in charge of each fields. Fields were then refined and validated by experts. Literature analysis and formulation of recommendations were then performed using the Grading of Recommendations Assessment, Development and Evaluation (GRADE) system [8, 9]. A level of evidence was defined for each bibliographic reference as a function of the type of study. This level of evidence could be reassessed by considering the methodological quality of the study. The bibliographic references common to each outcome were then pooled. An overall level of evidence was determined for each outcome, considering the level of evidence of each bibliographic reference, the consistency of the results between the different studies, the direct or indirect nature of the evidences, cost analysis, and so forth. A “strong” level (Grade 1+ or 1−) of evidence enabled the formulation of a “strong” recommendation (i.e., should be done, should not be done) (Table 1). A “moderate,” “weak,” or “very weak” level of evidence (Grade 2+ or 2−) led to the drawing up of an “optional” recommendation (i.e., should probably be done, should probably not be done). When the evidence was lacking, a recommendation resulting from “expert opinion” could be formulated. The proposed recommendations were then presented to and discussed with the whole panel of experts. The aim was not necessarily to reach a single and convergent opinion of the experts regarding all proposals but to bring out the points of agreement and disagreement or of indecision. Each recommendation was then evaluated and rated by each expert according to a scale from 1 (complete disagreement) to 9 (complete agreement). The collective score was established using a methodology derived from the RAND/UCLA Appropriateness Method [10]. After elimination of the extreme values (outliers), the median and terminals of the scores were calculated. The median defined disagreement between the experts when it was between 1 and 3, agreement between 7 and 9, and indecision between 4 and 6. The disagreement, agreement, or indecision was “strong” if the terminals were within the same range and “weak” if the terminals straddled two ranges. In the absence of strong agreement, the recommendations were reformulated and again scored with a view to achieving a better consensus. Two rounds of scoring were therefore performed.

**Table 1:** Evidence grading and recommendations formulation

Risk of bias and grade	Type of recommendation	Formulation
*Low: Grade 1*	Positive recommendation +	Should be
High level of evidence	Negative recommendation −	Should not be
*Intermediate to high: Grade 2*	Positive recommendation +	Should probably be
Intermediate to low level of evidence	Negative recommendation −	Should probably not be
*High: expert opinion*	Positive recommendation +	Should probably be (expert opinion)
No available data	Negative recommendation −	Should probably not be (expert opinion)

A panel of pediatricians reviewed the pediatric literature. In the absence of available evidence or pediatric specificity, adult recommendations apply to the pediatric patients. In case of pediatric specificity, a dedicated recommendation was planned to be added, supported by an argumentative text and with specific validation by the pediatrician panel.

## Results and recommendations

### Scope of the recommendations

The present recommendations apply to neutropenia of all etiologies, except for constitutive, hereditary, newborn autoimmune-related neutropenia, and transient sepsis-induced neutropenia. Considering a higher infectious risk during profound neutropenia (PMN less than 500/mm^3^), they apply particularly to these patients. They also apply to patients with non-functional PMN (functional neutropenia) and neutropenia expected in the coming 48 h.

Six fields were predefined:

I. ICU admission and prognosis
II. Protective isolation and prophylaxis
III. Acute respiratory failure (ARF)
IV. Organ failure and support
V. Antibiotic management and source control (except for invasive fungal infection)
VI. Hematological management (hematopoietic growth factors and transfusions, except for platelet transfusions evaluated in previous recommendations) [11].

The experts in charge of the fifth field considered, as regards to the lack of specific data in ICU patients regarding antifungal management, either for empirical or for curative treatment that this point was outside the scope of the current recommendations. The experts agreed that usual recommendations (http://www.kobe.fr/ecil/publications.htm) should apply to critically ill neutropenic patients. In the same line, since specific recommendations exist regarding management of febrile neutropenia [2], only specificities of critically ill neutropenic patients were reported in this statement.

Seventeen experts and one coordinator participated as per these recommendations. A bibliographic review, including publications in French and English, was done in the PubMed and Cochrane databases. A specific analysis of the pediatric literature was also performed.

Forty recommendations were formulated, including a specific pediatric recommendation (Table 2). Among them, 7 were strong recommendations, 28 were weak recommendations, and 5 resulted from expert opinion. Twenty-six (65 %) recommendations obtained a strong agreement.

**Table 2:** Summary of expert recommendations

*I. ICU admission and prognosis*
RI-1—Neutropenia should probably not be used as triage criteria in cancer patients considered for ICU admission. Performance status, comorbidities, and potentially life-prolonging treatment available are more relevant in this regard (Grade 2−, strong agreement)
RI-2—Neutropenia should probably not be considered as a prognostic factor in critically ill cancer patients (Grade 2-, weak agreement)
RI-3−Intensive care unit admission should probably not be delayed if ICU admission is deemed necessary in critically ill cancer patients (Grade 2-, strong agreement)
*II. Prophylaxis and protective isolation*
RII-1—Protective isolation should probably be considered in patients with profound (neutrophil count less than 500/mm^3^) and prolonged (expected neutropenia duration more than 7 days) neutropenia (Grade 2+, strong agreement)
RII-2—Protective isolation should not be considered as a sterile isolation (Grade 1-, strong agreement)
RII-3—Protective isolation should not delay ICU admission or limit patients’ clinical monitoring or access to patients’ rooms in cases of emergency (Grade 1-, strong agreement).
RII-4—Antibacterial prophylaxis should probably not be performed in critically patients with neutropenia (Grade 2-, strong agreement)
RII-5—Anti-Aspergillus prophylaxis should probably be used in critically ill neutropenic patients with acute myeloid leukemia or myelodysplastic syndrome with both induction and consolidation therapy used when neutropenia is expected to be profound (neutrophil count less than 500/mm^3^) and with an expected duration of at least 15 days (Grade 2+, weak agreement)
RII-6—Anti-Aspergillus prophylaxis should probably be used in high-risk critically ill neutropenic patients (myeloablative conditioning regimens, older patients, transplant in patients with active disease, umbilical/placental cord blood transplant) (Grade 2+, weak agreement)
RII-7−Anti-Aspergillus prophylaxis should probably be used in critically ill neutropenic patients with severe idiopathic medullary aplasia (neutrophil count less than 500/mm^3^) (Grade 2+, weak agreement)
*III. Acute respiratory failure management*
RIII-1—Acute respiratory failure should be considered as a therapeutic emergency in critically ill patients with neutropenia (Grade 1+, strong agreement)
RIII-2—Etiological diagnosis of ARF should be considered as a primary objective in this setting (Grade 1+, strong agreement)
RIII-3—The diagnostic workup should include systematic analysis of the underlying condition, severity and duration of neutropenia, underlying immunosuppression, preexisting treatment and prophylaxis, clinical course of ARF, and clinical and radiological features (Grade 1+, strong agreement)
RIII-4—Invasive and non-invasive diagnostic tests should probably be prescribed according to pretest probability rather than being performed systematically. This should particularly be the case for bronchoscopy with bronchoalveolar lavage (Grade 2+, strong agreement)
RIII-5—Pulmonary biopsies should probably be performed only on a case-by-case basis by a multidisciplinary team after careful assessment of both clinical suspicion and the risk-to-benefit ratio (Grade 2+, strong agreement)
*IV. Organ failure and organ support*
RIV-1—Neutropenic enterocolitis (typhlitis) should probably be considered in critically ill neutropenic patients with fever and acute abdomen, particularly in cases of recent cancer chemotherapy known to be associated with a high rate of oral or gastrointestinal toxicity (Grade 2+, strong agreement)
RIV-2—In adult patients, a complete diagnostic workup, including an abdominal CT scan with contrast media, should probably be performed (Grade 2+, strong agreement). In the pediatric setting, abdominal ultrasonography should probably be performed as first-line imaging (Grade 2+, strong agreement)
RIV-3—First-line colonoscopy should probably be avoided in patients with high suspicion of typhlitis (expert opinion, strong agreement)
RIV-4—Management of typhlitis should include broad-spectrum antibiotic therapy along with multidisciplinary management, including consultation of a general or abdominal surgeon (Grade 1+, strong agreement)
RIV-5—Neutropenia and thrombocytopenia should not modify the timing of surgery in patients with suspicion of digestive tract perforation (Grade 1+, strong agreement)
RIV-6—Neutropenia in itself should probably not modify ventilatory support in critically ill cancer patients (Grade 2−, strong agreement)
RIV-7—Invasive mechanical ventilation should probably not be delayed only as a consequence of neutropenia, underlying malignancy, or immunocompromised status (Grade 2−, weak agreement)
RIV-8—An indication for renal replacement therapy should probably not be modified by neutropenia in itself (Grade 2−, strong agreement)
*V. Antibacterial therapy and source control management*
RV-1—Combination therapy with aminoglycoside should probably be used as initial antibiotic therapy in neutropenic patients with severe sepsis or septic shock (expert opinion, weak agreement)
RV-2—Glycopeptide antibiotic adjunctive agents (or other agents active against resistant aerobic gram-positive cocci) should probably be considered for the following specific clinical indications
V-2-a—Suspected catheter-related infection (Grade 2+, strong agreement)
V-2-b—Skin or soft tissue infection (Grade 2+, strong agreement)
V-2-c—Severe sepsis or septic shock (Grade 2+, weak agreement)
V-2-d—Use of antipseudomonal -lactam agent with insufficient anti-gram-positive activity (ceftazidime, for example) (Grade 2+, weak agreement)
V-2-e—Grade III or IV mucositis (Grade 2+, weak agreement)
V-2-f−Known colonization with methicillin-resistant Staphylococcus aureus (Grade 2+, weak agreement)
RV-3—If used empirically, glycopeptide antibiotics should probably be reconsidered and discontinued in the following situations
After 72 h and if no resistant gram-positive cocci have been identified (expert opinion, weak agreement)
If infection is related to bacteria susceptible to a -lactam agent (expert opinion, strong agreement)
RV-4—Antibiotic de-escalation should probably be considered in the following situations
When infection is related to susceptible organism (expert opinion, strong agreement)
In patients without documented bacterial infection and with stable clinical condition (expert opinion, weak agreement)
RV-5—Indwelling catheters should probably be removed immediately in neutropenic patients with septic shock and no identifiable clinical infection (Grade 2+, strong agreement)
*VI. Hematological management*
RVI-1—Prophylactic use of G-CSF should probably be initiated or resumed in critically ill patients with neutropenia or requiring cancer chemotherapy with expected medullary toxicity (Grade 2+, weak agreement)
RVI-2—G-CSF should probably be stopped when worsening of respiratory status during neutropenia recovery is suspected or before neutropenia recovery in patients at high risk of worsening of respiratory status during neutropenia recovery (preexisting respiratory failure or pulmonary infection) (Grade 2+, strong agreement)
RVI-3—In euvolemic patients, transfusion of packed red blood cells should probably be performed according to a restrictive strategy in such a way as to maintain hemoglobin above a 7 g/dl threshold (Grade 2+, strong agreement)
RVI-4—Granulocyte transfusion should probably not be performed as adjunct therapy in neutropenic critically ill patients with severe infection (Grade 2−, strong agreement)

### ICU admission and prognosis

#### **RI-1**

Neutropenia should probably not be used as triage criteria in cancer patients considered for ICU admission. Performance status, comorbidities, and potentially life-prolonging treatment available are more relevant in this regard (Grade 2−, strong agreement).

#### **RI-2**

Neutropenia should probably not be considered as a prognostic factor in critically ill cancer patients (Grade 2−, weak agreement).

#### **RI-3**

Intensive care unit admission should probably not be delayed if ICU admission is deemed necessary in critically ill cancer patients (Grade 2−, strong agreement).

The prognostic impact of neutropenia remains controversial. Hence, neutropenia remains a transient and expected immune dysfunction. Although neutropenia is an independent risk factor for poor outcome in the general ICU population with severe sepsis or septic shock [6] and in earlier cohorts of critically ill cancer patients [12, 13], several recent studies failed to demonstrate any impact of neutropenia, neutropenia duration, or resolution of neutropenia on the outcome of critically ill cancer patients [7, 14–24]. Although this finding remains in an unselected population of neutropenic critically ill patients, it must be noted that specific infectious disease such as *Fusarium* infection is associated with a nearly constant mortality in the absence of neutropenia recovery [25].

In addition, early ICU admission should probably be considered in this subgroup of patients. Hence, delayed ICU admission ranging from hours to days has been independently associated with increased mortality [26–30]. Although the studies in this field were at high risk of bias, this finding, along with the high rate of secondary ICU admission in patients initially considered “too well” to benefit from ICU treatment [31], prompted experts to recommend early ICU admission of these patients.

### Prophylaxis and protective isolation

#### **RII-1**

Protective isolation should probably be considered in patients with profound (neutrophil count less than 500/mm^3^) and prolonged (expected neutropenia duration more than 7 days) neutropenia (Grade 2+, strong agreement).

#### **RII-2**

Protective isolation should not be considered as a sterile isolation (Grade 1−, strong agreement).

#### **RII-3**

Protective isolation should not delay ICU admission or limit patients’ clinical monitoring or access to patients’ rooms in cases of emergency (Grade 1−, strong agreement).

Most of the studies assessing the benefits of protective isolation were performed more than two decades ago, in ward patients, and are at a high risk of bias. In these studies, full protective isolation (including geographic isolation, technical isolation, high-efficiency air filtration, and digestive decontamination) proved to be efficient in patients with profound and prolonged neutropenia with regard to the rate of infection [32, 33], severe infection [32–36], induction failure, or mortality rate [32, 36, 37]. The benefit of protective isolation seems to be correlated with neutropenia severity, although the impact of functional neutropenia has never been assessed, to the best of our knowledge [32]. Most of the studies performed with partial protective isolation, however, failed to demonstrate any benefit [34, 37, 38]. Besides full protective isolation, benefits of high-efficiency air filtration have been underlined in several studies performed before the validation of antifungal prophylaxis protocols [39–41].

With regard to the available evidence, protective isolation might be of interest in patients with profound (neutrophil count less than 500/mm^3^) and prolonged neutropenia (expected duration more than 7 days). Ideally, this protective isolation includes the following:

1. High-efficiency air filtration [filtration of 99.7 % of particles greater than or equal to 0.3 µm; International Organization for Standardization (ISO) class 5 or better]
2. Geographic isolation
3. Technical isolation, including a face mask and a cap

Other measures (including gloves or digestive decontamination) were found to be of debatable interest.

The benefits of protective isolation in patients with expected neutropenia shorter than 7 days or in patients with neutrophil counts greater than 500/mm^3^ are uncertain.

Studies performed in other settings, however, suggest that isolation decreases the quality of care, limits access to a patient’s room, and increases the risk of severe and severe avoidable adverse events [42, 43]. With regard to the limited quality of studies validating protective isolation in the ward, the lack of studies dedicated to critically ill cancer patients, and the high risk of decreased access to instable patients consecutive to protective isolation, the experts strongly believe that protective isolation should not delay ICU admission, limit patients’ clinical monitoring, or limit access to patients’ rooms in cases of emergency.

Additional studies are warranted to confirm preexisting studies and validate potential benefits in ward or specific settings such as critical care.

#### **RII-4**

Antibacterial prophylaxis should probably not be performed in critically patients with neutropenia (Grade 2−, strong agreement).

Several studies and a meta-analysis have validated the benefits of antibacterial prophylaxis in patients with profound and prolonged neutropenia [44–46]. Quinolones with and without gram-positive bacterial activity and co-trimoxazole have been validated in this regard [46]. These studies and a meta-analysis underlined the benefits in terms of the risk of fever and documented bacterial infection, as well as mortality [46]. However, routine use of these strategies was associated with an increased rate of bacterial resistance [46–49]. These studies were performed in non-critically ill patients [46]. To the best of our knowledge, no study to date has validated such antibacterial prophylaxis strategies in ICU settings, and no data are available to assess ecological risk with regard to bacterial resistance. The risk-to-benefit ratio of such prophylactic strategies is therefore unknown. Although additional studies are warranted, experts believe that bacterial prophylaxis should probably not be used in this setting.

#### **RII-5**

Anti-Aspergillus prophylaxis should probably be used in critically ill neutropenic patients with acute myeloid leukemia or myelodysplastic syndrome with both induction and consolidation therapy used when neutropenia is expected to be profound (neutrophil count less than 500/mm^3^) and with an expected duration of at least 15 days (Grade 2+, weak agreement).

#### **RII-6**

Anti-Aspergillus prophylaxis should probably be used in high-risk critically ill neutropenic patients (myeloablative conditioning regimens, older patients, transplant in patients with active disease, umbilical/placental cord blood transplant) (Grade 2 + , weak agreement).

#### **RII-7**

Anti-Aspergillus prophylaxis should probably be used in critically ill neutropenic patients with severe idiopathic medullary aplasia (neutrophil count less than 500/mm^3^) (Grade 2+, weak agreement).

Acute respiratory failure in patients with fungal infection remains associated with a grim outcome [50]. Although specific data in critically ill patients are lacking, the risk of aspergillosis is closely correlated with the severity and duration of neutropenia, with a significantly higher risk in patients with profound neutropenia during 15 days or more, which may occur during the induction or intensive consolidation of acute myeloid leukemia/myelodysplastic syndrome or during a myeloablative regimen of an allogeneic stem cell transplantation. In this setting, two large randomized controlled trials (RCTs) with low levels of bias have validated anti-*Aspergillus* prophylaxis using posaconazole [51, 52]. These studies demonstrated a decreased rate of fungal infection [51, 52] as well as decreased overall [51] and fungal mortality [52]. Similarly, anti-aspergillosis has been recommended in patients with severe idiopathic medullary aplasia [53]. Although specific data in critically ill patients are lacking, experts have selected to recommend the use of anti-*Aspergillus* prophylaxis in these specific subgroups of patients should they require ICU admission. Although these specific situations require prophylaxis, several uncertainties remain in ICU patients among which bioavailability deserves to be mentioned. Thus, optimal dosage, administration scheme, formulation remain to be assessed in this specific population of patients [54].

Other subgroups of critically ill patients with neutropenia may require anti-*Aspergillus* prophylaxis on a case-by-case basis. In these subgroups, the help of an attending hematologist may be required.

Similarly, specific antiviral and *Pneumocystis* prophylaxis may be required according to underlying malignancy and previous treatments. These prophylaxes should probably be used according to the usual indications even in critically ill patients.

### Acute respiratory failure

#### **RIII-1**

Acute respiratory failure should be considered as a therapeutic emergency in critically ill patients with neutropenia (Grade 1+, strong agreement).

#### **RIII-2**

Etiological diagnosis of ARF should be considered as a primary objective in this setting (Grade 1+, strong agreement).

#### **RIII-3**

The diagnostic workup should include systematic analysis of the underlying condition, severity and duration of neutropenia, underlying immunosuppression, preexisting treatment and prophylaxis, clinical course of ARF, and clinical and radiological features (Grade 1+, strong agreement).

Acute respiratory failure remains a frequent complication of immunocompromised and neutropenic patients [22, 55]. Early management is associated with decreased mortality and might help in increasing the safety of diagnostic procedures such as bronchoscopy [28, 55]. Although several infectious and non-infectious etiologies might be involved and might be intricate in these patients [55–63], a systematic diagnostic workup may lead to a high-probability diagnosis in 70–80 % of patients with ARF [55–63]. ARF of unknown origin is associated with a grim prognosis, suggesting diagnosis to be a major goal of management of these patients [56–58, 64].

Several studies validated a non-invasive and invasive systematic diagnostic workup that may explain the increasing rate of high-probability diagnoses in this setting [56–58, 64]. This strategy includes a systematic analysis of the underlying malignancy and mechanism of immunosuppression, received treatment and prophylaxis, clinical and radiological picture, and clinical course of the respiratory failure [58]. This analysis is the first step in allowing the assessment of pretest probability and organization of a diagnostic test strategy. This pretest probability is mandatory to allow the interpretation of subsequent invasive and non-invasive test results. The reader may find more specific information regarding diagnostic workup in this setting in previous review [56, 65].

#### **RIII-4**

Invasive and non-invasive diagnostic tests should probably be prescribed according to pretest probability rather than being performed systematically. This should particularly be the case for bronchoscopy with bronchoalveolar lavage (Grade 2+, strong agreement).

Bronchoscopy with bronchoalveolar lavage (BAL) remains diagnostic in up to two-thirds of ARF cases in immunocompromised patients and is associated with therapeutic changes in 30–50 % of patients [57, 60, 64, 66–84]. In addition, rate of respiratory deterioration in the hours following BAL is substantial, one-third of patients without invasive mechanical ventilation experiencing significant respiratory worsening [57, 64]. In one RCT, researchers compared diagnostic strategies in critically ill patients without invasive mechanical ventilation. These strategies were an invasive strategy with systematic BAL and a non-invasive strategy including a first-line non-invasive test (Additional file 1) along with subsequent BAL in patients without a diagnosis or in those requiring mechanical ventilation [64]. In that study, the need for mechanical ventilation, patient mortality, and the rate of patients without a diagnosis after the diagnostic workup were similar to both strategies [64]. Although these results suggest that BAL was well tolerated, they also challenge the diagnostic benefits of first-line BAL when compared with a non-invasive diagnostic workup. BAL remains mandatory in cases of failure to obtain a diagnosis non-invasively or if deemed necessary to validate a clinical or radiological suspicion (e.g., infiltrative pulmonary disease, alveolar proteinosis). In the same line, pretest probability should be the primary trigger in drawing optimal diagnostic workup rather than patients’ condition (with vs. without MV for example) or associated conditions (thrombocytopenia for example). When required, the rate of clinical worsening following BAL in patients requiring mechanical ventilation remains limited [57, 85].

#### **RIII-5**

Pulmonary biopsies should probably be performed only on a case-by-case basis by a multidisciplinary team after careful assessment of both clinical suspicion and the risk-to-benefit ratio (Grade 2+, strong agreement).

After a negative diagnostic workup and BAL are performed, transbronchial biopsies might be helpful in selected patients. They can be performed only after the control of bleeding risk. The complication rate is high, however, with pneumothorax occurring in 4–20 % of the patients, which is higher in patients requiring mechanical ventilation [78, 86–88].

Transparietal needle biopsies guided by either computed tomography or ultrasonography may be helpful in patients with nodular and peripheral lesions [89–93].

Lastly, open-lung or video-assisted thoracoscopic lung biopsies can be performed in neutropenic patients under mechanical ventilation [94–96]. The latter should probably be performed when other diagnostic strategies have been exhausted after failure to obtain a diagnosis. When performed, these biopsies should probably include both pathological and extensive microbiological examination. However, the diagnostic rate, particularly in cases of diffuse lung disease, is limited.

With regard to the high rate of complications, these diagnostic strategies should be discussed by multidisciplinary staff, the risk-to-benefit ratio should be carefully assessed, and these biopsies should ideally be performed in high-volume centers.

### Organ failure and organ support

#### **RIV-1**

Neutropenic enterocolitis (typhlitis) should probably be considered in critically ill neutropenic patients with fever and acute abdomen, particularly in cases of recent cancer chemotherapy known to be associated with a high rate of oral or gastrointestinal toxicity (Grade 2+, strong agreement).

Typhlitis usually occurs after cancer chemotherapy associated with a high rate of oral or gastrointestinal toxicity (high-dose cytarabine for example). Clinical manifestations of typhlitis include fever, abdominal cramping, abdominal distention, occlusion, pain and/or tenderness, diarrhea, and intestinal bleeding; however, they are relatively non-specific. In addition, several conditions encountered in neutropenic patients and including pseudomembranous colitis, digestive graft-versus-host disease (GVHD), viral infections, or acute surgical abdomen may mimic typhlitis. Standardized diagnostic criteria have been proposed by Groschlüter et al. [97]. These criteria include the presence of profound neutropenia (neutrophil count less than 100/mm^3^), bowel wall thickening greater than 4 mm in a computed tomographic (CT) examination of any segment of the bowl at least 30 mm length, and the exclusion of other diagnoses, such as *Clostridium difficile*-associated colitis, GVHD, or other abdominal syndromes.

#### **RIV-2**

In adult patients, a complete diagnostic workup, including an abdominal CT scan with contrast media, should probably be performed (Grade 2+, strong agreement). In the pediatric setting, abdominal ultrasonography should probably be performed as first-line imaging (Grade 2+, strong agreement).

#### **RIV-3**

First-line colonoscopy should probably be avoided in patients with high suspicion of typhlitis (expert opinion, strong agreement).

Systematic diagnostic workup should be performed in patients with abdominal symptom compatible with typhlitis following chemotherapy associated with a high rate of oral or gastrointestinal toxicity. Systematic abdominal CT scans with contrast enhancement may help in diagnostic confirmation and allow a search for complications such as perforations. In the pediatric setting, CT scans may be used as a second-line examination in cases of suspected perforation and if ultrasonography is inconclusive [98–100]. In this setting, the feasibility and reliability of ultrasonography in measuring bowel wall thickness has been validated, with typhlitis being likely when bowel wall thickness measured by ultrasonography is greater than 3 mm [100]. In addition, the radiation dose associated with CT in the pediatric setting is important [101], and the use of ultrasonography may help in avoiding sedation, which is frequently required for CT scans.

Along with abdominal imaging, the diagnostic workup should include blood and stool cultures along with a search for *C. difficile* toxin. Although colonoscopy or recto-sigmoidoscopy may be required as second-line diagnostic test, these examinations are associated with a high risk of perforation in patients with suspected typhlitis [102].

#### **RIV-4**

Management of typhlitis should include broad-spectrum antibiotic therapy along with multidisciplinary management, including consultation of a general or abdominal surgeon (Grade 1+, strong agreement).

#### **RIV-5**

Neutropenia and thrombocytopenia should not modify the timing of surgery in patients with suspicion of digestive tract perforation (Grade 1+, strong agreement).

In cases of typhlitis, conservative management is preferred. This treatment includes symptomatic measures such as maintenance of fluid and electrolyte balance, antalgic treatment, and monitoring. A nasogastric tube may be required in cases of ileus, and parenteral nutrition may be required in cases of severe typhlitis. Use of granulocyte colony-stimulating factor (G-CSF) remains of uncertain benefit, and its routine use cannot be recommended.

Use of broad-spectrum antibiotic therapy remains the rule and should be adapted to local microbiological ecology and patients’ colonization [103]. Ideally, this treatment should be active on *Enterococcus*, *Enterobacteriaceae*, anaerobes, and *Pseudomonas aeruginosa* [2, 103]. The systematic use of glycopeptide or metronidazole is of uncertain benefit but has been advocated by some authors [2, 103]. First-line empiric antifungal therapy cannot be recommended, owing to the low incidence of invasive fungal infection (5 %) during typhlitis [104]. Nevertheless, lack of clinical improvement within 72 h should prompt antifungal therapy initiation [104, 105]. Antibiotics are usually stopped after neutropenia and clinical recovery.

Surgical management may be required and may be prompted by meeting the following objective criteria: uncontrolled bleeding despite transfusion, suspicion of gastrointestinal perforation, and clinical worsening despite conservative management [106]. Sequential abdominal imaging may be required and may help in assessing changes in bowel wall thickness. Ultrasonography may help in this regard [107–110], and decreases in bowel wall thickness on sequential ultrasonography have been associated with clinical improvement [108]. In contrast, mortality remains high (60 vs. 4 %; *P* < 0.001) in patients with bowel wall thickness greater than 10 mm [111]. Aggressive surgical management in these patients should probably be discussed on a case-by-case basis.

Although surgical management requires a multidisciplinary assessment, several factors need to be taken into account. (1) First, inadequately treated typhlitis carries a high risk of death [112]. (2) In the more severe patients with typhlitis, lack of surgical management was found to be a significant adverse prognostic factor [113]. (3) Data obtained in non-cancer patients with thrombocytopenia suggest that even high-risk hemorrhage surgical intervention such as splenectomy carries a low risk of morbidity or mortality [114]. Taken together, these data suggest that neutropenia or thrombocytopenia *per se* should not lead to delayed surgical intervention in critically ill neutropenic patients suspected of digestive tract perforation.

#### **RIV-6**

Neutropenia in itself should probably not modify ventilatory support in critically ill cancer patients (Grade 2−, strong agreement).

#### **RIV-7**

Invasive mechanical ventilation should probably not be delayed only as a consequence of neutropenia, underlying malignancy, or immunocompromised status (Grade 2−, weak agreement).

The need for mechanical ventilation has been associated with a nearly constant mortality rate in cohort studies performed two decades ago or more [23, 115]. In addition, earlier cohort studies and small RCTs suggested non-invasive mechanical ventilation to be associated with a decreased rate of invasive mechanical ventilation and mortality in critically ill cancer patients with ARF [72, 116–118]. However, findings regarding the benefits of prophylactic non-invasive ventilation were inconsistent [119], and recent cohort studies pointed out decreased mortality in patients requiring invasive mechanical ventilation [120–122]. A recent randomized trial failed to demonstrate any benefits of non-invasive ventilation when compared with oxygen therapy in immunocompromised patients [123]. With regard to available evidence, systematic use of non-invasive ventilation in immunocompromised patients no longer seems justified.

It must be noted that no study was specifically focused on pediatric patients. Additional studies in this setting are therefore required.

#### **RIV-8**

An indication for renal replacement therapy should probably not be modified by neutropenia in itself (Grade 2−, strong agreement).

Although the need for renal replacement therapy (RRT) remains associated with a poor outcome in critically ill cancer patients, several recent observational studies demonstrated an overall mortality close to that for the general ICU population [7, 124]. Except for specific indications such as tumor lysis syndrome, the indications for RRT are similar to those in the general ICU population.

## Antibacterial therapy and source control management

### **RV-1**

Combination therapy with aminoglycoside should probably be used as initial antibiotic therapy in neutropenic patients with severe sepsis or septic shock (expert opinion, weak agreement).

A systematic review of the literature failed to identify evidence with a low risk of bias in critically ill neutropenic patients. Neutropenic critically ill patients with fever or suspicion of sepsis should be considered at high risk of severe infection and receive an empirical antibiotic therapy including an antipseudomonal β-lactam agent with activity against gram-positive bacteria [2]. In these patients, systematic use of combination therapy with aminoglycosides remains speculative. In a systematic review focused on the general neutropenic patient population, Paul et al. [125] found no benefit of combination therapy in terms of mortality. In addition, combination therapy was associated with an increased rate of renal toxicity [125]. Little information was available with regard to the more severe cases, however. Two low-level evidence studies specifically demonstrated a potential benefit of aminoglycosides in critically ill patients with neutropenia and severe sepsis and/or septic shock [126, 127]. Although experts therefore recommend using combination therapy in this specific subgroup, they also underline the need for studies producing high-grade evidence in this setting.

### **RV-2**

Glycopeptide antibiotic adjunctive agents (or other agents active against resistant aerobic gram-positive cocci) should probably be considered for the following specific clinical indications:

- V-2-a–Suspected catheter-related infection (Grade 2+, strong agreement).
- V-2-b–Skin or soft tissue infection (Grade 2+, strong agreement).
- V-2-c–Severe sepsis or septic shock (Grade 2+, weak agreement).
- V-2-d–Use of antipseudomonal β-lactam agent with insufficient anti-gram-positive activity (ceftazidime, for example) (Grade 2+, weak agreement).
- V-2-e–Grade III or IV mucositis (Grade 2+, weak agreement).
- V-2-f–Known colonization with methicillin-resistant Staphylococcus aureus (Grade 2+, weak agreement).

Studies dedicated to critically ill neutropenic patients in this setting are lacking. Systematic reviews performed in the general population of neutropenic patients suggest a lack of efficacy and potential toxicity of glycopeptide antibiotics when used systematically as an adjunct to an antipseudomonal β-lactam agent [128, 129]. Although systematic use of these molecules cannot be advocated, several areas of uncertainty remain, including potential benefits in patients with more severe illness or in specific subgroups. Combination therapy including glycopeptide antibiotics or other agents active against resistant aerobic gram-positive cocci should probably be considered in patients with more severe illness, patients at high risk of typhlitis (i.e., patients with severe mucositis), and patients at high risk of resistant gram-positive bacterial infection.

### **RV-3**

If used empirically, glycopeptide antibiotics should probably be reconsidered and discontinued in the following situations:

- After 72 h and if no resistant gram-positive cocci have been identified (expert opinion, weak agreement).
- If infection is related to bacteria susceptible to a β-lactam agent (expert opinion, strong agreement).

### **RV-4**

Antibiotic de-escalation should probably be considered in the following situations:

- When infection is related to susceptible organism (expert opinion, strong agreement).
- In patients without documented bacterial infection and with stable clinical condition (expert opinion, weak agreement).

Antibiotic de-escalation has been advocated in the general population of neutropenic patients [2]. Nevertheless, high-grade evidence is lacking in the general population and in critically ill neutropenic patients. A single, prospective, monocentric study found that de-escalation could be performed in 44 % of neutropenic critically ill patients with antibiotic therapy, this de-escalation being well tolerated in both the short and long term [130].

Microbiological cultures should be performed in critically ill neutropenic patients [64]. Bacterial identification may help in de-escalating antibiotic therapy, limiting exposure to wide-spectrum therapy and therefore limiting the risk of subsequent multidrug-resistant bacterial infection or colonization [130, 131]. Empirical use of anti-gram-positive antibiotic therapy should be associated with a search for methicillin-resistant *S. aureus* colonization. The sensitivity of this screening approaches 100 % [132]. Should the results of microbiological cultures and colonization screening for resistant gram-positive bacteria be negative, empirical therapy should probably be reconsidered and in most of the clinical scenarios discontinued. Among receiving antipseudomonal β-lactam agents [2], de-escalation in patients with susceptible organisms or stable clinical condition may be considered and seems safe [130]. Although the experts advocated de-escalation in above-mentioned situations, high-quality evidences are needed in this field.

### **RV-5**

Indwelling catheters should probably be removed immediately in neutropenic patients with septic shock and no identifiable clinical infection (Grade 2+, strong agreement).

Immediate indwelling catheter removal has been independently associated with increased survival in critically ill neutropenic patients and patients with severe sepsis and/or septic shock [126, 133]. Although additional studies may be required, experts advocate a liberal removal strategy in this subgroup of patients with severe illness.

## Hematological management

### **RVI-1**

Prophylactic use of G-CSF should probably be initiated or resumed in critically ill patients with neutropenia or requiring cancer chemotherapy with expected medullary toxicity (Grade 2+, weak agreement).

Prophylactic use of G-CSF in patients with hematological malignancy or solid tumors has proven efficacy in decreasing the risk or duration of neutropenia and limiting the risk of infectious disease [134–136]. In meta-analyses performed in patients with non-Hodgkin’s lymphoma or solid tumors, use of G-CSF was associated with a decrease in both overall mortality and infection-related mortality [135–137]. Little data on this topic in the ICU are available, suggesting limited efficacy in this setting [72, 138], and it was obtained from studies with low-level evidence. Thus, despite uncertainties and while additional studies are needed, it seems reasonable to recommend using prophylactic G-CSF when available or deemed necessary by an oncologist or hematologist.

Conversely, use of G-CSF in patients with already overt infections (curative G-CSF) was found to have a limited benefit in neutropenic patients [139, 140]. In addition, only limited data exist in the ICU, and this treatment remains associated with potential side effects, including risk of worsening respiratory status. Experts were unable to recommend use of curative G-CSF, and this treatment should be used only on a case-by-case basis with an attending oncologist or hematologist.

### **RVI-2**

G-CSF should probably be stopped when worsening of respiratory status during neutropenia recovery is suspected or before neutropenia recovery in patients at high risk of worsening of respiratory status during neutropenia recovery (preexisting respiratory failure or pulmonary infection) (Grade 2+, strong agreement).

Worsening of respiratory status may occur during the 3-day period surrounding neutropenia recovery [141]. Both clinical [142–145] and experimental [146, 147] studies suggest G-CSF to be associated with respiratory worsening in this setting. Although all of these reports are of low-level evidence, they suggest that, along with the remaining uncertainties regarding G-CSF benefits in ICU patients, G-CSF should be stopped in high-risk patients in the days preceding neutropenia recovery.

### **RVI-3**

In euvolemic patients, transfusion of packed red blood cells should probably be performed according to a restrictive strategy in such a way as to maintain hemoglobin above a 7 g/dl threshold (Grade 2+, strong agreement).

No specific study has been performed in neutropenic critically ill patients. However, several observational studies suggest an independent association between transfusion of packed red blood cells and increased mortality rate and risk of nosocomial infection. A recent meta-analysis including 18 randomized studies (7593 patients) suggested a decrease in risk of nosocomial infection with a restrictive transfusion strategy [148]. In patients with euvolemic anemia and without acute coronary syndrome, available high-grade evidence suggests a restrictive strategy with the aim of maintaining a hemoglobin threshold between 7 and 9 g/dl to limit the need for transfusion without an increase in mortality or cardiac events compared with a liberal strategy [149, 150]. Experts believe that these results should be applied to neutropenic patients.

### **RVI-4**

Granulocyte transfusion should probably not be performed as adjunct therapy in neutropenic critically ill patients with severe infection (Grade 2−, strong agreement).

A recent systematic review of the benefits of granulocyte transfusion in neutropenic patients with infection was inconclusive with regard to the limited number of studies available and high heterogeneity [151]. In addition, patients included in most of the studies had profound and prolonged neutropenia. A recent RCT failed to demonstrate any benefit of granulocyte transfusion, although the study was clearly underpowered to detect such an effect [152]. While the experts acknowledge the lack of adequately powered studies and believe additional studies are required, they believe that the remaining doubts, along with potential deleterious effects of granulocyte transfusion, justify limiting this therapeutic option to clinical research.

## Additional file

10.1186/s13613-016-0189-6 Non exhaustive list of non invasive test that may be considered for diagnostic of acute respiratory failure.
