# Supplementary material for: Management of neutropenic patients in the intensive care unit (NEWBORNS EXCLUDED) recommendations from an expert panel from the French Intensive Care Society (SRLF) with the French Group for Pediatric Intensive Care Emergencies (GFRUP), the French Society of Anesthesia and Intensive Care (SFAR), the French Society of Hematology (SFH), the French Society for Hospital Hygiene (SF2H), and the French Infectious Diseases Society (SPILF)
Source: Ann Intensive Care. 2016 Sep 15;6:90. doi: 10.1186/s13613-016-0189-6 (PMC5025409; doi:10.1186/s13613-016-0189-6)
Supplement: Supplementary file 1 — 10.1186/s13613-016-0189-6 Non exhaustive list of non invasive test that may be considered for diagnostic of acute respiratory failure. [file 13613_2016_189_MOESM1_ESM.docx]

**MANAGEMENT OF NEUTROPENIC PATIENTS IN THE INTENSIVE CARE UNIT**

**(NEWBORNS EXCLUDED)**

**Recommendations from an expert panel from the French Intensive Care Society (SRLF) with the French Group for Pediatric Intensive Care Emergencies (GFRUP), the French Society of Anesthesia and Intensive Care (SFAR), the French Society of Hematology (SFH), the French Society for Hospital Hygiene (SF2H), and the French Infectious Diseases Society (SPILF)**

David SCHNELL et al.

Supplementary appendix

**S1- Non-exclusive list of non-invasive test that may be considered for acute respiratory failure diagnosis [reproduced from Azoulay et al. Am J Respir Crit Care Med 2010; Manuscript reference #60]**

**1. Imaging**

Chest radiograph

High-resolution computed tomography

**2. Echocardiography**

**3. Sputum examination** for Bacteria, Candida spp., other fungi, and Tuberculosis

**4. Induced sputum** (P. jiroveci)

**5. Nasopharyngeal aspirates** for viral purposes

**6. Blood cultures**

**7. Polymerase chain reaction** test for Herpes viridae and Cytomegalovirus

**8. Circulating Aspergillus galactomanan**

**9. Serologic tests** for Chlamydiae pneumonia, Mycoplasma pneumonia, Legionella pneumophila

**10. Urine antigen** for Legionella pneumophila and Streptococcus pneumoniae
